# Bacteria identified from deep nasopharyngeal swabs and non-endoscopic bronchoalveolar lavage in calves on farms with a history of bronchopneumonia

**DOI:** 10.1186/s13028-026-00861-w

**Published:** 2026-03-14

**Authors:** Jasmin Laschinger, Joachim Spergser, Benedikt Taxacher, Thomas Wittek, Katharina Lichtmannsperger

**Affiliations:** 1 Clinical Department for Farm Animals and Food System Science, Clinical Center for Ruminant and Camelid Medicine, University of Veterinary Medicine Vienna, Veterinaerplatz 1, 1210 Vienna, Austria; 2 Department of Biological Sciences and Pathobiology, Centre of Pathobiology, University of Veterinary Medicine Vienna, Veterinaerplatz 1, 1210 Vienna, Austria

**Keywords:** Beef cattle, Bovine respiratory disease, Culture, *Mycoplasma bovis*, PCR

## Abstract

**Background:**

We describe the occurrence of bacteria associated with bovine respiratory disease (BRD) on Austrian beef operations and compare bacterial culture results from double guarded deep nasopharyngeal swab (DNS) and non-endoscopic bronchoalveolar lavage (BAL) samples. Fifty pre-weaned beef calves from 13 conveniently selected commercial beef operations with a history of BRD were sampled between April and August 2024. From each calf a DNS and a BAL sample was obtained and cultured for *Pasteurella multocida*, *Mannheimia haemolytica*, *Histophilus somni*, *Bibersteinia trehalosi*, *Mannheimia* spp. and *Mycoplasma bovis*. Additionally, a *M. bovis* specific PCR was carried out on all samples. The objectives of this study were to describe and compare the occurrence of bacteria in DNS and BAL samples from Austrian beef calves on beef operations with a history of BRD, and to determine the agreement between *M. bovis* culture and PCR test results in these samples.

**Results:**

In total, 21 bacterial isolates were obtained from 15 of the 50 DNS samples and 22 isolates from 17 of the 48 BAL samples. *P. multocida*, *Ma. haemolytica*, *H. somni*, *B. trehalosi* and *Mannheimia* spp. were isolated from 4, 2, 5, 0, and 3 DNS samples, and from 4, 3, 3, 2, and 1 BAL samples, respectively. *M. bovis* was cultured most frequently, i.e. from 7/50 DNS samples and 9/48 BAL samples. All PCR positive BAL (21/48) samples showed a PCR positive DNS (26/50) as well. Of the PCR positive samples, *M. bovis* was cultured in 27% of DNS samples and in 43% of BAL samples. A consistent culture result was found in 4/48 calves from both DNS and BAL samples, with *M. bovis* identified in all cases. The study showed moderate agreement for *M. bovis* culture (κ = 0.45) and almost perfect agreement for PCR (κ = 0.88) between DNS and BAL, respectively, but poor agreement for other bacteria (κ=-0.91 – -0.03).

**Conclusions:**

Common BRD-associated bacteria occur on beef operations in Austria even in calves with mild BRD signs. Additionally, BAL and DNS are suitable for bacterial isolation from the respiratory tract and can provide an overview of the BRD-associated bacteria involved on the farm. We detected a poor agreement between the results of bacterial isolation from DNS and BAL samples in this study, except for *M*. *bovis*.

## Background

Bovine respiratory disease (BRD) in cattle is a common disease of young calves resulting in a high morbidity and mortality in affected herds [1]. A recent study from Austria showed that 39.8% of the dairy farmers report an incidence of BRD of > 10% [2].

BRD is caused by a wide range of viral and bacterial pathogens and involves several host-environment-pathogen interactions [1]. *Mannheimia* (*Ma*.) *haemolytica*, *Pasteurella* (*P*.) *multocida*, *Histophilus* (*H*.) *somni* and *Mycoplasma* (*M*.) *bovis* are among the bacterial pathogens most frequently detected [1, 3]. These bacterial pathogens are commonly isolated from the upper and lower respiratory tracts of both clinically healthy and diseased cattle, highlighting their widespread occurrence and potential role as opportunistic pathogens in the development of BRD [4]. Despite remaining controversial in some scientific communities and countries, *M*. *bovis* is widely accepted as a primary pathogen [5]. Isolation of bacteria through culture, rather than PCR analysis, is crucial for performing susceptibility testing to ensure appropriate use of antimicrobials [4]. Since 2024, veterinarians in Austria are forced by law to perform antimicrobial susceptibility testing in case of repeated or prolonged use of antimicrobials on the farms which is the case on multiple farms with a history of BRD [6]. It has been described that guarded and non-guarded deep nasopharyngeal swabs (DNS), transtracheal aspiration (TTA), and endoscopic or non-endoscopic bronchoalveolar lavage (BAL) can be used for sampling the respiratory tract in cattle [7]. In addition, *post-mortem* lung tissue can be directly sampled during necropsies. Sampling techniques differ in terms of the technical effort and time requirement, the costs and the degree of invasiveness and risk, e.g., haemorrhage, emphysema from performing a TTA, for the sampled animals [4]. In fattening bulls, a moderate level of agreement (κ = 0.50) has been described for single guarded DNS and TTA culture results for *Ma. haemolytica* [8]. Compared to non-guarded DNS, bronchoalveolar lavage samples are less likely to have multiple bacteria growth, more likely to be negative, and produce more single bacteria cultures [9]. A previous study [9] described that 79.2% of calves with BRD have a clinically interpretable culture result from BAL samples, compared to only 31.2% of the DNS samples. Calves in the aforementioned study were categorized as suffering from BRD based on the assessment of clinical signs and lung ultrasound findings. Frequencies of bacterial isolation were lower in healthy calves compared to diseased calves, but did not differ between DNS and BAL samples in either group. The only species more likely to be isolated from BAL samples was *H*. *somni* [9]. Other authors detected only moderate agreement between single guarded DNS and endoscopic BAL [3, 10] with kappa values between 0.47 ± 0.17 to 0.61 ± 0.23 depending on the bacterium [10]. The paired BAL and lung tissue cultures from necropsied cattle showed perfect agreement [11]. However, in a different study, mycoplasmas found in the lower respiratory tract were not detected by nasal cultures from non-guarded swabs [11]. In contrast, isolates from the deep nasopharynx, which are sampled using a double guarded DNS, are highly representative of the isolates found in the lung for *Ma*. *haemolytica* and *M*. *bovis* [12].

The main objectives of the present study were (1) to describe the herd-level and calf-level occurrence of bacteria in DNS and BAL samples from calves on beef operations with a history of BRD (2) to compare the outcome of bacterial isolation between double guarded DNS and non-endoscopic BAL samples in Austrian beef calves and (3) to determine the agreement between the detection of *M*. *bovis* culture and the PCR test results in these samples. We hypothesized that DNS and non-endoscopic BAL produce identical bacteriology results and that the agreement between *M*. *bovis* culture and *M*. *bovis* PCR is high.

## Methods

### Farms and animals

Thirteen commercial beef operations located in Lower Austria or Salzburg, whose local veterinarians had contacted the University Ruminant Clinic for a problem-solving herd health visit due to recurring BRD cases, were visited between April and August 2024 as part of the clinic’s herd health management consultancy. The farms were located within a radius of 250 km from the University of Veterinary Medicine Vienna. The calves were procured from various sources, including auctions (72.0%), cattle traders (26.0%), and directly from other farms (2.0%), with an age between 18 and 137 days (median = 66 days; first quartile (Q1) = 50 days; third quartile (Q3) = 81 days). The included calves were housed in straw bedded pens of 4 to 84 animals on beef finishing units, where bought-in calves are managed all under one roof. The farm size was between 70 and 400 cattle. Except for one calf, all calves were male and had been purchased for beef finishing between 1 and 71 days prior to the herd visit. A total of 36 calves were sampled within one week after purchase, four calves between one and three weeks, and ten calves more than three weeks after purchase.

During the herd health visit a minimum of three and a maximum of five pre-weaned beef calves per farm with clinical signs of BRD or preselected by the farmer underwent a short physical examination and sampling. The scoring system described in the literature [13] was used to assess the severity of BRD-associated clinical signs, i.e. nasal discharge, coughing, depression, and rectal temperature, in individual calves. Each clinical sign was scored individually, and the scores were summed to obtain a cumulative score per calf, with a maximum possible score of 12. Calves with a total score of ≤ 1, 2 or 3, and ≥ 4 were classified as mild (*n* = 31), moderate (*n* = 16) and severe (*n* = 3) cases, respectively. The median clinical score was 1 (Q1 = 0; Q3 = 2). Calves within a current withdrawal period from previous treatment using antimicrobial drugs, as well as calves previously vaccinated against BRD were excluded. This procedure resulted in a convenience sample of 50 calves.

### DNS and BAL sampling

The sampling procedure was performed on the non-sedated, standing calf with their head held straight and slightly over-extended using a commercial halter and the nostrils were disinfected with 70% ethanol and a swab. From each calf, a double guarded DNS and a BAL sample was drawn.

A sterile 76 cm double guarded culture swab (EQUIVET; Jørgen Kruuse A/S; Denmark) was introduced medioventrally in the nasal cavity approximately 2 cm rostral to the medial canthus of the eye. Then the inner tube was advanced approximately 1 cm and thereafter the protected swab was also advanced approximately 1 cm until the nasopharyngeal tissue was reached. After rotating several times, the swab was retracted into the inner protecting tube, the inner tube retracted into the outer tube, and the whole equipment was taken out. The swab was placed in a sterile 15 mL Greiner tube containing 5 mL 2SP transport medium (0.2 mol/L sucrose in 0.02 mol/L phosphate buffer comprising a mixture of mono- and dibasic potassium phosphate, pH 7.0 ± 0.2, supplemented with 10% fetal calf serum).

Bronchoalveolar lavage fluid was collected by using the Easy Lavage Set (iVet^®^; Quidee GmbH; Germany). The sampling catheter was advanced into the tube up to the valve tip. Subsequently, the syringe, which was filled with 40 mL sterile sodium chloride solution, was attached. The catheter was inserted medioventrally in the nasal cavity, passed through the larynx and trachea, and gently advanced until the superior thoracic aperture was reached. Thereafter, 30–35 mL of 0.9% NaCl was injected into the bronchi and immediately aspirated, with the recovery of at least 10% of the fluid being targeted. If no fluid was recovered, a second 20 mL injection was attempted. Sample quality was verified by inspecting for the presence of the characteristic foam layer, indicating contact with surfactant. The aspirated lavage fluid was transferred to a sterile cup. For each calf, a new, sterile tube and catheter was used. The sampling procedures were performed by one of the authors. All samples were cooled between + 2 °C to + 8 °C during transport in a polystyrene box to the laboratory and analysed within 24 h after sampling. BAL sampling was not performed on two calves due to the unavailability of sufficient sterile tracheal tubes during one farm visit.

### Microbiological examination

For the cultural detection of bacteria, the DNS and 100 µL of BAL samples were plated onto Columbia III agar with 5% sheep blood (BD Diagnostics, Vienna, Austria) using the three-phase streaking method. Plates were incubated micro-aerobically at 37 °C under 6% CO2 atmosphere for 48 h. Microbial growth was semi-quantitatively graded as sporadic, slight, moderate, or heavy depending on the occurrence and number of isolated colonies in streaking sections. Bacterial isolates were identified to the species level (score values ≥ 2.00) or the genus level (scores ≥ 1.80–1.99) using matrix-assisted laser desorption ionization – time of flight (MALDI-ToF) mass spectrometry as described previously [14]. For the isolation of mycoplasmas, 100 µL of 2SP (DNS) and 100 µL of BAL sample were plated onto PPLO agar (BD Diagnostics, Vienna, Austria) supplemented with 20% (v/v) horse serum and 25% (v/v) yeast extract and incubated at 37 °C under 6% CO2 atmosphere for up to 10 days. Plates were daily checked for mycoplasma growth using a stereomicroscope. If typical mycoplasma colonies were observed, they were picked and transferred into 5 mL PPLO broth medium (BD Diagnostics, Vienna, Austria) supplemented with 20% (v/v) horse serum, 25% (v/v) yeast extract, and 2% (w/v) sodium pyruvate. Culture media with picked colonies were incubated at 37 °C in ambient air until a colour change of the medium occurred (usually after 2–3 days of incubation) and cultures were subsequently identified by MALDI-ToF mass spectrometry [15].

For the molecular detection of *M*. *bovis*, 1 mL each of the 2SP medium (DNS) and BAL samples were centrifuged at 20,000 x g for 10 min and DNA extracted from the pellets using the DNeasy Blood & Tissue kit (Qiagen, Hilden, Germany). Subsequently, a *M*. *bovis* specific endpoint PCR was performed targeting the *polC* gene as previously described [16].

### Data analysis

The data obtained from the physical examination on the farm and microbiology results were analyzed descriptively using Microsoft Excel 2020 (Microsoft Corp., Redmond, WA, USA) and IBM^®^ SPSS^®^ Statistics Version 29 (IBM^®^, New York, USA). No sample size or power calculation was conducted prior to the study, meaning that it is based on a convenient sample. The Kappa statistic was calculated to determine the agreement between DNS and BAL regarding the isolation of *P*. *multocida*, *Ma*. *haemolytica*, *H*. *somni*, *B*. *trehalosi*, *Mannheimia* spp. and *M*. *bovis* in culture, as well as *M*. *bovis* culture and PCR results from DNS and BAL samples [17]. The level of agreement for the kappa coefficient (κ) was classified as follows: ≤0 indicates poor agreement, 0.10–0.20 indicates slight agreement, 0.21–0.40 indicates fair agreement, 0.41–0.60 indicates moderate agreement, 0.61–0.80 indicates substantial agreement, and 0.81–1.0 indicates almost perfect agreement [18]. *P* < 0.05 was considered statistically significant.

## Results

### Herd-level occurrence

Four herds yielded negative results in culture and PCR (herd 8, 9, 11 and 12). In total, 9 of the 13 herds (69.2%) showed a positive culture result for *P*. *multocida*, *Ma*. *haemolytica*, *H*. *somni*, *B*. *trehalosi*, *Mannheimia* spp. (non-*Ma*. *haemolytica* isolates of the genus *Mannheima* that could not be identified to species level using MALDI-ToF MS) or *M*. *bovis*. Seven of the 13 farms (53.9%) were positive in DNS and 9 of the 13 (69.2%) farms in the BAL. *P*. *multocida*, *Ma*. *haemolytica*, *H*. *somni* and *Mannheimia* spp. were cultured from 38.5%, 53.8%, 15.4% and 23.1% of farms, respectively (Table 1). *B*. *trehalosi* was cultured from none of the 13 farms using DNS samples but from 2 of the 13 farms using the BAL samples. PCR for *M*. *bovis* was positive in 61.5% DNS vs. 53.8% BAL samples of herds, whereas in 53.8% of herds this bacterium was cultured from DNS or BAL samples.

**Table 1:** Bacterial cultures and PCR results of DNS and BAL samples from beef calves (13 herds)

			Number (percentage) of positive cultures for:												Number (Percentage) of positive PCR for:	
Herd	Age range (days)	Calves (*n*)	Pasteurella multocida		Mannheimia haemolytica		Histophilus somni		Bibersteinia trehalosi		Mannheimia spp.		Mycoplasma bovis		Mycoplasma bovis	
			DNS	BAL	DNS	BAL	DNS	BAL	DNS	BAL	DNS	BAL	DNS	BAL	DNS	BAL
1	35 ─ 60	5	0 (0.0)	0 (0.0)	0 (0.0)	1 (20.0)	0 (0.0)	0 (0.0)	0 (0.0)	0 (0.0)	1 (20.0)	0 (0.0)	0 (0.0)	0 (0.0)	0 (0.0)	0 (0.0)
2	56 ─ 92	5	2 (40.0)	1 (20.0)	1 (20.0)	0 (0.0)	0 (0.0)	0 (0.0)	0 (0.0)	0 (0.0)	0 (0.0)	0 (0.0)	1 (20.0)	2 (40.0)	3 (60.0)	3 (60.0)
3	65 ─ 144	4	0 (0.0)	1 (25.0)	0 (0.0)	1 (25.0)	0 (0.0)	1 (25.0)	0 (0.0)	1 (25.0)	1 (25.0)	1 (25.0)	1 (25.0)	1 (25.0)	4 (100.0)	4 (100.0)
4	80 ─ 115	5	1 (20.0)	1 (20.0)	0 (0.0)	0 (0.0)	0 (0.0)	0 (0.0)	0 (0.0)	1 (20.0)	0 (0.0)	0 (0.0)	0 (0.0)	1 (20.0)	5 (100.0)	5 (100.0)
5	63 ─ 81	4	0 (0.0)	0 (0.0)	1 (25.0)	0 (0.0)	2 (50.0)	2 (50.0)	0 (0.0)	0 (0.0)	0 (0.0)	0 (0.0)	1 (25.0)	3 (75.0)	4 (100.0)	4 (100.0)
6	37 ─ 93	5	1 (20.0)	0 (0.0)	0 (0.0)	1 (20.0)	0 (0.0)	0 (0.0)	0 (0.0)	0 (0.0)	0 (0.0)	0 (0.0)	1 (20.0)	1 (20.0)	3 (60.0)	2 (40.0)
7	46 ─ 67	4	0 (0.0)	1 (25.0)	0 (0.0)	0 (0.0)	0 (0.0)	0 (0.0)	0 (0.0)	0 (0.0)	0 (0.0)	0 (0.0)	1 (25.0)	1 (25.0)	3 (75.0)	2 (50.0)
8	35 ─ 52	3	0 (0.0)	0 (0.0)	0 (0.0)	0 (0.0)	0 (0.0)	0 (0.0)	0 (0.0)	0 (0.0)	0 (0.0)	0 (0.0)	0 (0.0)	0 (0.0)	0 (0.0)	0 (0.0)
9	72 ─ 89	3	0 (0.0)	0 (0.0)	0 (0.0)	0 (0.0)	0 (0.0)	0 (0.0)	0 (0.0)	0 (0.0)	0 (0.0)	0 (0.0)	0 (0.0)	0 (0.0)	0 (0.0)	0 (0.0)
10	76 ─ 89	3 | 1^#^	0 (0.0)	0 (0.0)	2 (66.7)	0 (0.0)	0 (0.0)	0 (0.0)	0 (0.0)	0 (0.0)	1 (33.3)	0 (0.0)	2 (66.7)	0 (0.0)	3 (100.0)	1 (100.0)
11	42 ─ 53	3	0 (0.0)	0 (0.0)	0 (0.0)	0 (0.0)	0 (0.0)	0 (0.0)	0 (0.0)	0 (0.0)	0 (0.0)	0 (0.0)	0 (0.0)	0 (0.0)	0 (0.0)	0 (0.0)
12	30 ─ 39	3	0 (0.0)	0 (0.0)	0 (0.0)	0 (0.0)	0 (0.0)	0 (0.0)	0 (0.0)	0 (0.0)	0 (0.0)	0 (0.0)	0 (0.0)	0 (0.0)	0 (0.0)	0 (0.0)
13	51 ─ 77	3	0 (0.0)	0 (0.0)	1 (33.3)	0 (0.0)	0 (0.0)	0 (0.0)	0 (0.0)	0 (0.0)	0 (0.0)	0 (0.0)	0 (0.0)	0 (0.0)	1 (33.3)	0 (0.0)
Total farms*			3* (23.1)	4* (30.8)	4* (30.8)	3* (23.1)	1* (7.7)	2* (15.4)	0* (0.0)	2* (15.4)	3* (23.1)	1* (7.7)	6* (46.2)	6* (46.2)	8* (61.5)	7* (53.8)

*DNS* double guarded deep nasopharyngeal swab, *BAL* non-endoscopic bronchoalveolar lavage

n = Number of calves sampled per farm = 100%

* = Total number of farms where the respective bacterial pathogen was detected in at least one calf

# = BAL sampling was not performed on two calves due to the unavailability of sufficient sterile tracheal tubes during one farm visit

### Calf-level occurrence

Concerning DNS samples, a BRD-associated bacterium (i.e., *P*. *multocida*, *M*. *haemolytica*, *H*. *somni*, *B*. *trehalosi*, *Mannheimia* spp., *M*. *bovis*) was isolated in a total of 30.0% (15/50), with two different bacteria (*M*. *bovis* and either *Mannheimia* spp. or *Ma*. *haemolytica*) being cultured in two calves and three different bacteria (*Ma*. *haemolytica*, *M*. *bovis* and either *P*. *multocida* or *H*. *somni*) in two calves. Overall, the most common bacteria were *M*. *bovis* (14.0%; 7/50) and *Ma*. *haemolytica* (10.0%; 5/50).

Similar results were obtained for BAL samples. A BRD-associated bacterium was detected in culture in 35.4% (17/48) of the samples, two different bacteria (*H*. *somni* and *M*. *bovis* in both cases) isolated in two calves and four different bacteria (*P*. *multocida*, *H*. *somni*, *B*. *trehalosi*, *M*. *bovis*) isolated in one calf. *M*. *bovis*, *H*. *somni* and *P*. *multocida* were the bacteria most commonly detected in BAL samples.

### Agreement between methods

In 73.1% (19/26) of the DNS samples, *M*. *bovis* was not detected in culture, but the PCR results were positive for *M*. *bovis* (κ = 0.26; Lower CI95 = 0.08; Upper CI95 = 0.44; *P* = 0.01). Fewer BAL samples than DNS samples yielded PCR positive results. In 57.1% (12/21) of BAL samples, *M. bovis* was detected by PCR, while culture results remained negative (κ = 0.46; Lower CI95 = 0.24; Upper CI95 = 0.68; *P* < 0.01). The highest level of agreement between DNS and BAL bacterial isolation was observed for *M*. *bovis*. All 21 calves showing a positive BAL PCR result also showed a positive DNS PCR result (Table 2). The DNS and BAL culture results matched only for *M*. *bovis* in four calves. Poor agreement for bacterial isolation from DNS and BAL was observed for all other bacteria.

Culture growth was rated as light, moderate and heavy growth. Cultures from BAL samples showed moderate to heavy bacterial growth more frequently (45.5%) than cultures from DNS (23.8%) (Table 3).

**Table 2:** Agreement of bacterial respiratory pathogen isolation between DNS (*n* = 50) and BAL (*n* = 48) samples in beef calves

	DNS (%)	BAL (%)	Kappa	95% CI (Lower CI; Upper CI)	*P* values
*P*. multocida (culture)	4 (8.0)	4 (8.0)	-0.09	-0.72; 0.54	0.53
*Ma. haemolytica* (culture)	5 (10.0)	3 (6.0)	-0.07	-0.60; 0.46	0.64
*H. somni* (culture)	2 (4.0)	3 (6.0)	-0.05	-0.10; -0.00	0.71
*B. trehalosi* (culture)	0 (0.0)	2 (4.0)	ND	ND	ND
*Mannheimia* spp. (culture)	3 (6.0)	1 (2.0)	-0.03	-0.08; -0.02	0.79
*M. bovis* (culture)	7 (14.0)	9 (18.8)	0.45	0.11; 0.79	0.00
*M. bovis* (PCR)	26 (52.0)	21 (43.8)	0.88	0.74; 1.01	0.00

*DNS* double guarded deep nasopharyngeal swabs, *BAL* non-endoscopic bronchoalveolar lavage

n = Number of calves sampled

ND = no statistical analysis can be conducted due to the insufficient number of observations in one of the groups

**Table 3:** Quantification of the respiratory bacteria isolated from DNS and BAL samples in beef calves

	DNS culture (*n* = 50)			BAL culture (*n* = 48)		
	Slight	Moderate	Heavy	Slight	Moderate	Heavy
*Pasteurella multocida*	4			3	1	
*Mannheimia haemolytica*	4	1		1	2	
*Histophilus somni*			2	1		2
*Bibersteinia trehalosi*				2		
*Mannheimia* spp.	3			1		
*Mycoplasma bovis*	5	1	1	4	2	3

*DNS* double guarded deep nasopharyngeal swabs, *BAL* non-endoscopic bronchoalveolar lavage

n = Number of calves sampled

Culture growth indicated from slight to heavy

## Discussion

In 69.2% of herds studied a positive culture result for at least one bacterium was obtained. However, there is considerable heterogeneity in the isolation rates of BRD-associated pathogens [7, 9–12]. Another study [11] obtained similar low isolation rates for *M*. *bovis* of 10% in unguarded nasal swabs and 29% at necropsy by flushing the cranial bronchi. In some studies, *H*. *somni* was not recovered from any of the samples [7, 12], which may be due to its fastidious growth requirements [4]. In contrast to a former study [7] we were able to isolate *B*. *trehalosi* from two BAL samples. This is a secondary bacterial pathogen in BRD and is isolated occasionally from cattle [4].

BRD-associated bacteria were isolated by culture 21 times in a total of 30.0% (15/50) of the DNS samples and 22 times in 35.4% (17/48) of BAL samples, with *M*. *bovis* being found most frequently. In the present study, *M*. *bovis* was detected in 18.8% of BAL samples and 14.0% of DNS samples. However, this numerical difference was not statistically evaluated. This is consistent with previous findings [11], which stated that BAL of the cranial bronchi is the most appropriate method to isolate *M*. *bovis* from cattle with BRD. Although not statistically tested, *H. somni* was numerically more often isolated from BAL fluids (6.0%) compared to DNS samples (4.0%). This is in accordance with an earlier report [9], in which this bacterium was isolated in 6.3% of BAL fluids compared to 1.4% of DNS samples.

In our study, 70.0% of the DNS and 64.6% of the BAL samples yielded a negative culture for BRD-associated bacteria (*P*. *multocida*, *M*. *haemolytica*, *H*. *somni*, *B*. *trehalosi*, *Mannheimia* spp., *M*. *bovis*). These values are higher compared to previous findings [9], in which 14.6% (21/144) of DNS and 40.3% (58/144) of BAL samples were negative in calves with BRD, and 12.8% (5/39) of DNS and 43.6% (17/39) of BAL samples were negative in healthy calves. However, that study did not include *B*. *trehalosi* and *Mannheimia* spp. in the analysis which might have led to false negative results. The lower isolation rates of BRD-associated bacteria in our study may therefore be explained by the selection of animals with mild BRD signs during the early stages of infection or the timing of the sample collection in relation to the progression of the disease. When sampling the lower respiratory tract during the initial phase of infection, it is possible that the bacterial load is insufficient for detection in bacterial culture [4]. Additionally, also the sensitivity and specificity of the culturing methods differ between the labs. Moreover, a large number of primary pathogens are viruses [1, 4], which were not included in the present study but may have been causing the BRD signs in the sampled calves. Parainfluenza-3 virus, bovine respiratory syncytial virus, bovine herpesvirus type 1, and bovine viral diarrhoea virus are considered primary viral respiratory pathogens [1], whereas Austria is currently free of the last two viruses [19]. These viruses can impair the host defence through various mechanisms, leading to bacterial pneumonia [1].

The high detection rate of *M*. *bovis* is surprising, as to the best of our knowledge there have been no studies that demonstrated the presence of these bacteria in calves in Austria. Nonetheless, a high detection rate was likely, considering the known high prevalence of this bacterium in Europe [20–22]. *M*. *bovis* is often considered as a primary bacterial pathogen associated with BRD [4, 22] and is intrinsically resistant to some first-line antibiotics like β-lactam antimicrobials and sulfonamides, and effectiveness of preventive vaccination is controversial [5, 23]. Therefore, we recommend Austrian farms to know their infection status for *M. bovis*. For Austrian beef operations, it may be advisable to include *M. bovis* in the diagnostic workup when facing herd problems with BRD, arthritis, or otitis. Sampling of the respiratory tract and pathogen identification by microbial culture or PCR are suitable for guiding immediate therapeutic decisions [4]. In contrast, serological testing for antibody detection is useful for informing vaccination strategies, assessing the immune status of animals, and evaluating infection dynamics at the herd level [4].

There was no dominant BRD bacterium isolated from calves within the same herd. The most likely explanation is that several calves were sampled one day after purchase and most of them originated from different farms, which indicates exposure to distinct bacteria. In addition, three to five calves per farm were sampled which had neither been previously vaccinated against BRD nor had been treated with antibiotics recently. Due to the small-scale farming structure of the farms and therefore the relatively low number of calves suffering severely from BRD at the same time, calves displaying mild clinical signs of BRD, or general ill thrift were also included. This might have impacted the results since the bacterial load was under the limit of detection, or viruses were the predominant pathogens at this early stage of the disease [1, 4]. However, due to the time lag between exposure to the pathogen and the onset of clinical signs [24], calves displaying only mild or no overt signs were also sampled. These animals were considered to be at high risk because they had recently been introduced to farms with a known history of BRD outbreaks. The majority of calves (n = 36) were sampled within one week after purchase, which may have been too early to detect pathogen spread within the group or herd, as previously noted. In contrast, other calves were sampled up to 71 days post-arrival, by which time any respiratory disease associated with introduction and mixing may have been successfully treated or resolved. Since local veterinarians had contacted the University Ruminant Clinic for problem-solving herd health visits due to recurring cases of BRD, we had limited influence over the timing of sampling, because each visit was scheduled as soon as possible following the veterinarian’ request.

Other authors enrolled calves with severe clinical signs defined as a score ≥ 5 and a rectal temperature of ≥ 39.4 °C [7]. In addition, no lung ultrasound was performed and therefore no further information on the degree of lung consolidation was available. Some studies recommend scanning the lungs to confirm ultrasonographic abnormalities and thus confirm BRD [25–27]. Another study, however, showed no significant differences in isolation rates or quantitative cultures between healthy calves, calves with upper respiratory tract infection, subclinical pneumonia and clinical pneumonia [28]. These findings support the assumption that positive bacterial culture results from lower respiratory samples do not necessarily indicate lung infection [3, 4, 28–30]. For differentiation of lower respiratory tract colonisation from infection in the presence of suggestive clinical signs and positive culture results, quantitative BAL bacterial counts have been suggested, but no clear cut-off values are available [28].

Sampling using double guarded DNS and non-endoscopic BAL is generally considered simple and time efficient [9]. Although achieving reliable BAL sampling requires a certain level of technical expertise, it can be successfully performed by less experienced veterinarians, provided they have received appropriate training or are working under supervision. The disadvantage of BAL is the higher costs of the equipment compared to DNS.

In the present study all calves were sampled while restrained using a halter without sedation and BAL was performed without endoscopic guidance. These procedure results in sampling a random lung lobe. In sedated calves dorsocaudal lung lobes are sampled, which are less frequently affected by BRD [31]. The advantage of an endoscopic BAL is that the most frequently affected cranial lobes can be sampled. Although it is still unclear, to what degree this variation in the BAL catheter’s intrapulmonary position influences the probability of isolating the bacteria [31].

Although, the results of detection of *M*. *bovis* in culture and PCR showed fair to moderate agreement for DNS and BAL samples, 73.1% of DNS and 57.1% of BAL samples showed a negative culture result despite positive PCR result. This result could be due to the difficulty of culturing mycoplasmas e.g. the need of specific media and slow growth rates [4]. In addition, PCR only detects the presence of the bacterium’s DNA, not its vitality. A false-positive PCR result can also be caused by contamination and leads to overdiagnosis of bacterial pneumonia [4, 28].

One limitation of this study is the small study population, which may reduce the validity and reliability of the results and should be taken into account when interpreting the findings. Since we did not carry out a sample size calculation/power calculation beforehand the kappa results with P values > 0.05 or negative kappa values need to be rejected. A retrospective sample size calculation suggested that a substantially larger number of samples would have been required to detect a statistically significant difference between groups with adequate power (desired power = 0.8). Therefore, the lack of statistical significance in some comparisons may be due to limited sample size rather than the absence of a true effect. Therefore, further investigations are needed where a power calculation is carried out in advance and that a certain number of positive results (higher prevalence of the bacteria) need to be included. Since the calves showed only slight clinical signs, the prevalence of BRD bacteria was not high resulting in non-significant and not valid kappa values, except for *M. bovis*. This results in a very limited external validity regarding the test comparison. The agreement, however, of *M*. *bovis* culture and PCR was high and statistically significant. In contrast, a previous study [9] found slight to moderate agreement between bacterial isolation of unguarded DNS and non-endoscopic BAL with kappa values between 0.16 and 0.58. These authors also calculated the best agreement for *M*. *bovis* isolation between non-guarded DNS and non-endoscopic BAL. Other studies reported moderate to substantial agreement for bacterial isolation of single guarded DNS and endoscopic BAL [10] and almost perfect agreement between TTA and unguarded nasal swabs, single guarded DNS or non-endoscopic BAL for the identification of *P*. *multocida*, *M*. *haemolytica* and *M*. *bovis* [7]. The results of these previous studies are highly variable. This may be due to different sampling methods (non-guarded/single guarded/double guarded DNS or endoscopic/non-endoscopic BAL with different equipment), alternating sampling sides of the nostrils, order of sampling, different definitions of cases, sampling times, transport/isolation/culturing method and for instance the experience of the operator. Moreover, the results of the present study may have been impacted by differences in the operators’ levels of experience with taking DNS or BAL samples, as well as variations in adherence to hygienic protocols that go along with these techniques. Although all operators were trained prior to the herd health visits, inconsistencies in execution – such as multiple attempts to enter the trachea or accidental entry into the oesophagus - may have increased the risk of BAL sample contamination [4, 9, 32]. The clinical signs and subsequently the prevalence of the bacteria was low. Therefore, the kappa values yielded negative, nonsignificant results. The limitations of this study include the relatively small sample size, the inclusion of calves with mild clinical signs of BRD due to the small-scale nature of the farms, the lack of ultrasound confirmation for pneumonia diagnosis, and the absence of healthy control groups. Furthermore, sampling was performed by multiple individuals. Although a standardized protocol was followed, minor variations in the procedure may have led to discrepancies in the results [4, 9, 32]. Another limitation was that the volume of aspirated lavage fluid was not measured, even though variations in the volume could have affected the quantity of target bacteria cultured [3, 9].

Studies have shown that post-mortem isolated bacteria from lung tissue show perfect agreement with bacteria isolated from BAL fluids obtained at necropsy by flushing the cranial bronchi of calves suffering from BRD [10]. The opposite applies for unguarded nasal swabs, which are not representative for bacterial pathogens isolated in the lung tissue [11]. Double guarded DNS, however, was found to be highly representative of the *Ma*. *haemolytica* and *M*. *bovis* isolates present in the lung [12]. When interpreting the results, it should be borne in mind that the analysis is based on a small sample size and that a large proportion of the animals had only mild clinical signs of BRD.

## Conclusions

The presented investigation showed that common BRD-associated bacteria occur on beef operations in Austria even in calves with mild BRD signs. Additionally, BAL and DNS are suitable for bacterial isolation from the respiratory tract and can provide an overview of the BRD-associated bacteria involved on the farm. This can be crucial for herd management regarding preventive and therapeutic measures for the herd. We found poor agreement between the results of bacterial isolation from DNS and BAL samples in this study, except for *M*. *bovis*. However, the sample size was small, and no power calculation was performed in advance therefore the study should be repeated in a broader setting.

## Data Availability

The datasets used and/or analyzed during the current study are available from the corresponding author on reasonable request.

## Declarations

### Prior publication

Data have not been published previously.
